# Ignorance and Infant Mortality

**Published:** 1899-03

**Authors:** 


### Ignorance and Infant Mortality.

Although the death rate of infants of the upper classes could certainly be greatly reduced by a more intelligent appreciation of the laws of health and particularly of those relating to diet, nevertheless it is among the very young of the lower classes that the most excessive mortality prevails. Ignorance and infant mortality go hand in hand. Hugh R. Jones, in his "Howard Medal Prize Essay," published in the journal of the Royal Statistical Society, says: "It is seen that diarrhea is exceedingly fatal during the first year of life—more especially during the earlier months. This fatality, in great measure, is due to the improper feeding of infants. Owing to the neglect and ignorance of mothers the infants are fed on contaminated milk or other food. Among the evidence presented by Dr. Jones, in his prize essay, tending to prove that infant mortality is largely due to ignorance, is a table from a report of the sanitary condition of Boston, Mass., in 1875, showing that in the New England and Middle States the infant mortality from cholera infantum and diarrhea was in proportion to the illiteracy. Dr. Vernon, Medical Officer of Health, for Southport, England, says: "My belief is that all the principal causes of infantile mortality are mostly results of ignorance—ignorance of the modes of spreading and means of restricting dangerous diseases; ignorance of the practical application of bacteriology and mycology to every-day affairs, particularly to food, water and food materials, on which human existence depends; ignorance of the effects of exposure of children to the ordinary atmospheric conditions; ignorance of that sort of knowledge which Herbert Spencer characterized as of "most worth"—knowledge which tends to directly preserve life; knowledge which it should be the function of the public school system to make paramount "because the life of the people is the supreme law." It is contended by some that infant mortality is ruled, to a very large extent, by the measure of the density of population. But that this is not wholly the case is shown by the fact that London, the largest of the towns in Great Britain, has also one of the smallest of infantile death rates. Therefore there must be other circumstances besides the conglomeration of people in towns to account for the excess of other large towns over the infant mortality of London. In pneumonia, now that it is known to be a communicable disease, much of the infantile mortality from this cause might be prevented if mothers and others in charge of children knew enough to take the necessary measures to prevent its spread. The same remarks apply to bronchitis and many maladies incidental to childhood. Dr. Henry M. Baker, the author of a very useful treatise on infantile mortality, in summing up, says with reference to infantile mortality: "The most important lessons which it is incumbent on me at this time to mention are: (1) It is astonishingly great; (2) it is most preventable; (3) it is due more to ignorance than to willfulness; (4) it is due to ignorance among the people of knowledge which is possessed by physicians who can make no general use of it for the benefit of the people except in some way they are permitted to teach it to them." As has been urged before in this journal, physicians should seize every opportunity to educate ignorant mothers how to care for their little ones.—*Pediatrics*.

